# The synthesis of ^15^N(7)-Hoogsteen face-labeled adenosine phosphoramidite for solid-phase RNA synthesis

**DOI:** 10.1007/s00706-016-1882-8

**Published:** 2016-12-08

**Authors:** Sandro Neuner, Christoph Kreutz, Ronald Micura

**Affiliations:** Institute of Organic Chemistry, Leopold-Franzens University, Innrain 80-82, Innsbruck, Austria

**Keywords:** Nucleosides, Modifications, RNA labeling, RNA structure, Base triplet, Twister ribozyme

## Abstract

**Abstract:**

We have developed an efficient route for the synthesis of ^15^N(7)-labeled adenosine as phosphoramidite building block for site- and atom-specific incorporation into RNA by automated solid-phase synthesis. Such labeled RNA is required for the evaluation of selected non-canonical base pair interactions in folded RNA using NMR spectroscopic methods.

**Graphical abstract:**

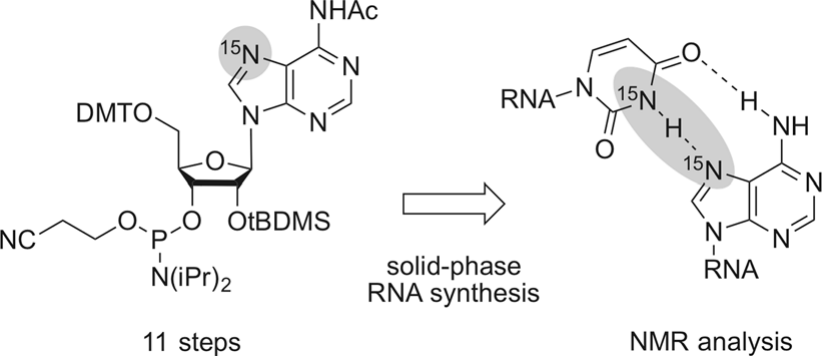

**Electronic supplementary material:**

The online version of this article (doi:10.1007/s00706-016-1882-8) contains supplementary material, which is available to authorized users.

## Introduction

Numerous reports on the synthesis of atom-specific ^15^N-labeled nucleosides exist in the literature; however, procedures for efficient access to the corresponding phosphoramidites for RNA solid-phase synthesis are rare [1–3]. We have recently described our preferred synthetic routes for ^15^N(1)-adenosine, ^15^N(1)-guanosine, ^15^N(3)-uridine, and ^15^N(3)-cytidine phosphoramidites which allow base pair-specific labeling in RNA for direct monitoring of Watson–Crick base pairs by ^1^H/^15^N/^15^N-COSY experiments [4]. The approach of individual Watson–Crick base pair labeling is particularly useful for the analysis of conformationally flexible RNAs when competing and interconverting secondary structures are encountered [5, 6].

Along the same line, the evaluation of more complex base pair interactions such as base triplets or quartets, and the underlying dynamics in solution is important to understand functional RNA structures. Thereby, selective nucleobase labeling is again advantageous for NMR spectroscopic investigations to directly spot the interaction of interest which in many cases involves the Hoogsteen face of one or more purine nucleosides. This is especially true for larger functional RNAs with complex folding, such as riboswitch aptamer domains and ribozymes, where spectral crowding can make the assignment procedure very labor-intensive or even impossible. Here, we present an optimized procedure to synthesize ^15^N(7)-labeled adenosine phosphoramidite. Additionally, a potential application to probe a *cis* Watson–Crick/Hoogsteen base pair interaction [7] is demonstrated for a base triplet that has been observed in the crystal structure of the *env22* twister ribozyme [8].

## Results and discussion

To achieve ^15^N(7)-labeled adenosine amidite **12**, we conceived a strategy that employs a silyl-Hilbert-Johnson nucleosidation [9–11] and a recently introduced azido-to-acetamido purine transformation [4] as key steps. Therefore, ^15^N(7)-hypoxanthine **5** was synthesized following the protocol by Jones and coworkers (Scheme 1) [12]. We started with sodium ethoxide-mediated cyclization of thiourea and ethyl cyanoacetate to form 6-amino-2-mercapto-pyrimidone (**1**) in high yields [13]. Nitrosylation of compound **1** installed the ^15^N-label by electrophilic substitution using the cost-effective isotope source Na^15^NO_2_ in aqueous acid. The deep red nitroso compound **2** precipitated and was directly reduced to the colorless diamino mercapto pyrimidone **3** with dithionite [12]. Subsequent desulfurization with activated nickel sponge in dilute aqueous ammonia yielded compound **4** [12]. Treatment with formic acid and diethoxymethyl acetate at elevated temperature resulted in formation of the imidazo moiety to furnish the desired ^15^N(7) hypoxanthine **5** [12]. In our hands, the 5-step reaction sequence proceeded in 75% overall yield and was conducted at multigram scales without the need for chromatographic purifications.

**Figure:**
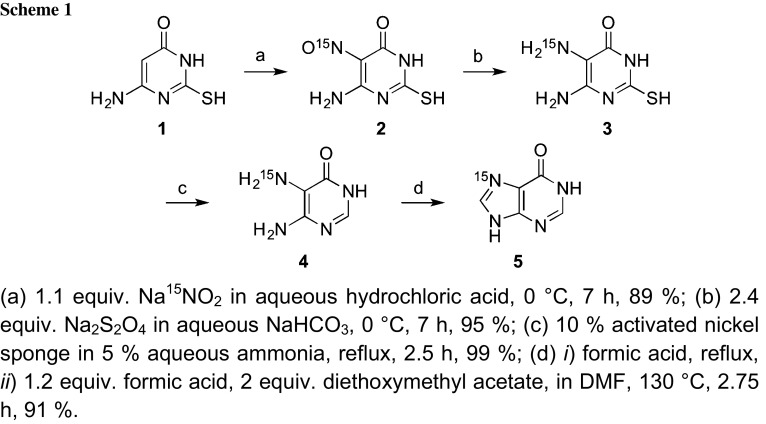

The nucleosidation reaction of ^15^N(7)-hypoxanthine **5** with 1-*O*-acetyl-2,3,5-tri-*O*-benzoyl-*ß*-d-ribofuranose under *Vorbrüggen* conditions proceeded in the presence of *N*,*O*-bis(trimethylsilyl)acetamide and trimethylsilyl triflate to give tribenzoylated ^15^N(7) inosine **6** as the major product, isolated after column chromatography in 56% yield. N1-, N3-, N7-, and/or O6-glycosylated isomers form as byproducts in minor amounts, consistent with previous reports [10, 11] and in accordance to the qualitative comparison of H-C8 ^1^H NMR resonances observed in the product mixture (Supplementary Material). The integrity (C1′–N9 connectivity) of compound **6** was further supported by NMR spectroscopic comparison of **6** with an authentic sample that was prepared by direct benzoylation of commercial inosine (Supplementary Material). Next, we activated the carbonyl group of compound **6** in analogy to a procedure that was originally developed by Wan and co-workers [14–16] for the mild activation of cyclic amides with (benzotriazol-1-yloxy)tris(dimethylamino)phosphonium hexafluorophosphate (BOP). Thereby, *O* ^6^-(benzotriazol-1-yl)inosine is formed which can be substituted by a nucleophilic displacement (*S*_N_Ar) reaction. These compounds can either be isolated [17] and used as convertible nucleosides after incorporation into DNA through solid-phase synthesis or directly derivatized with an appropriate electron-rich nucleophile [14–16]. For our route (Scheme 2), we used sodium azide to substitute the benzotriazolyl inosine intermediate. In the presence of Cs_2_CO_3_, this reaction proceeded smoothly in DMF to yield the 6-azido purine nucleoside **7** in 70% yield. Compound **7** was then reduced with thioacetic acid, resulting directly in the *N* ^6^-acetyl protected ^15^N(7) adenosine **8** in 84% yield. Then, selective deprotection of the ribose benzoyl groups was achieved in close to quantitative yield by treatment with aqueous NaOH in ethanol and pyridine. Functionalization of nucleoside **9** as building block for RNA solid-phase synthesis started with the introduction of a 4,4′-dimethoxytrityl group on the ribose 5′-OH to give compound **10** (59% yield), followed by *tert*-butyl-dimethylsilylation of the ribose 2′-OH to furnish compound **11** (51% yield). Finally, phosphitylation was executed with 2-cyanoethyl-*N*,*N*-diisopropylchlorophosphoramidite (CEP-Cl) in the presence of *N*,*N*-diisopropylethylamine in CH_2_Cl_2_. Starting with the nucleosidation reaction of ^15^N(7) hypoxanthine **6**, our route provides building block **12** in a 10% overall yield in seven steps and with six chromatographic purifications; in total, 1.6 g of **12** was obtained in the course of this study.

**Figure:**
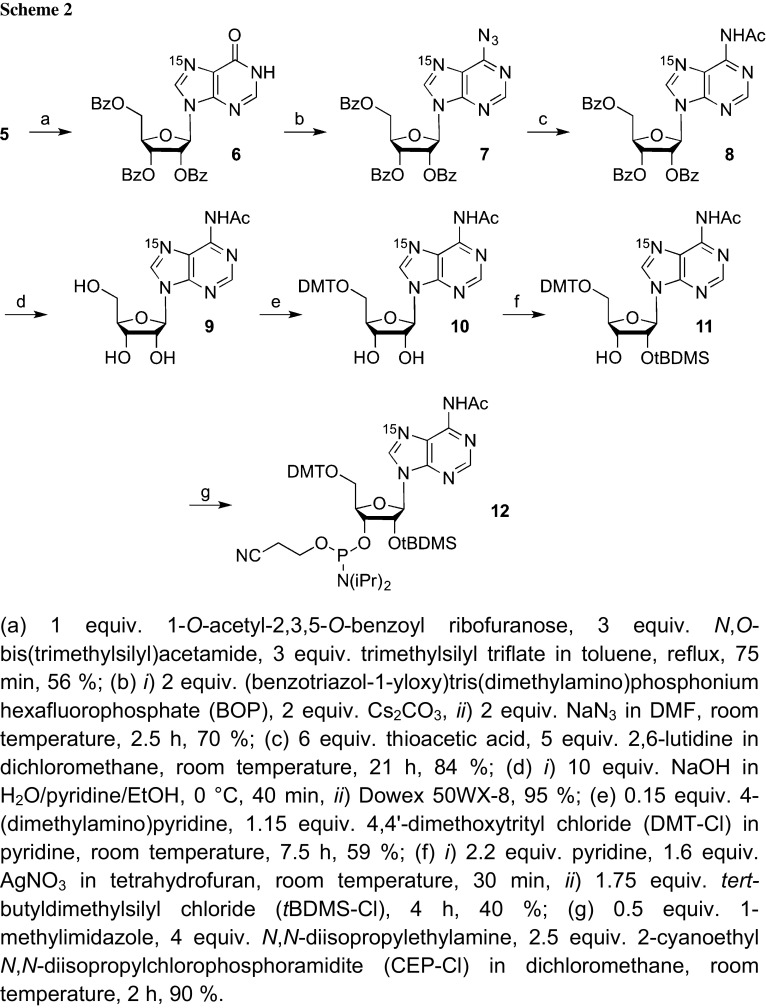

**Fig. 1:**
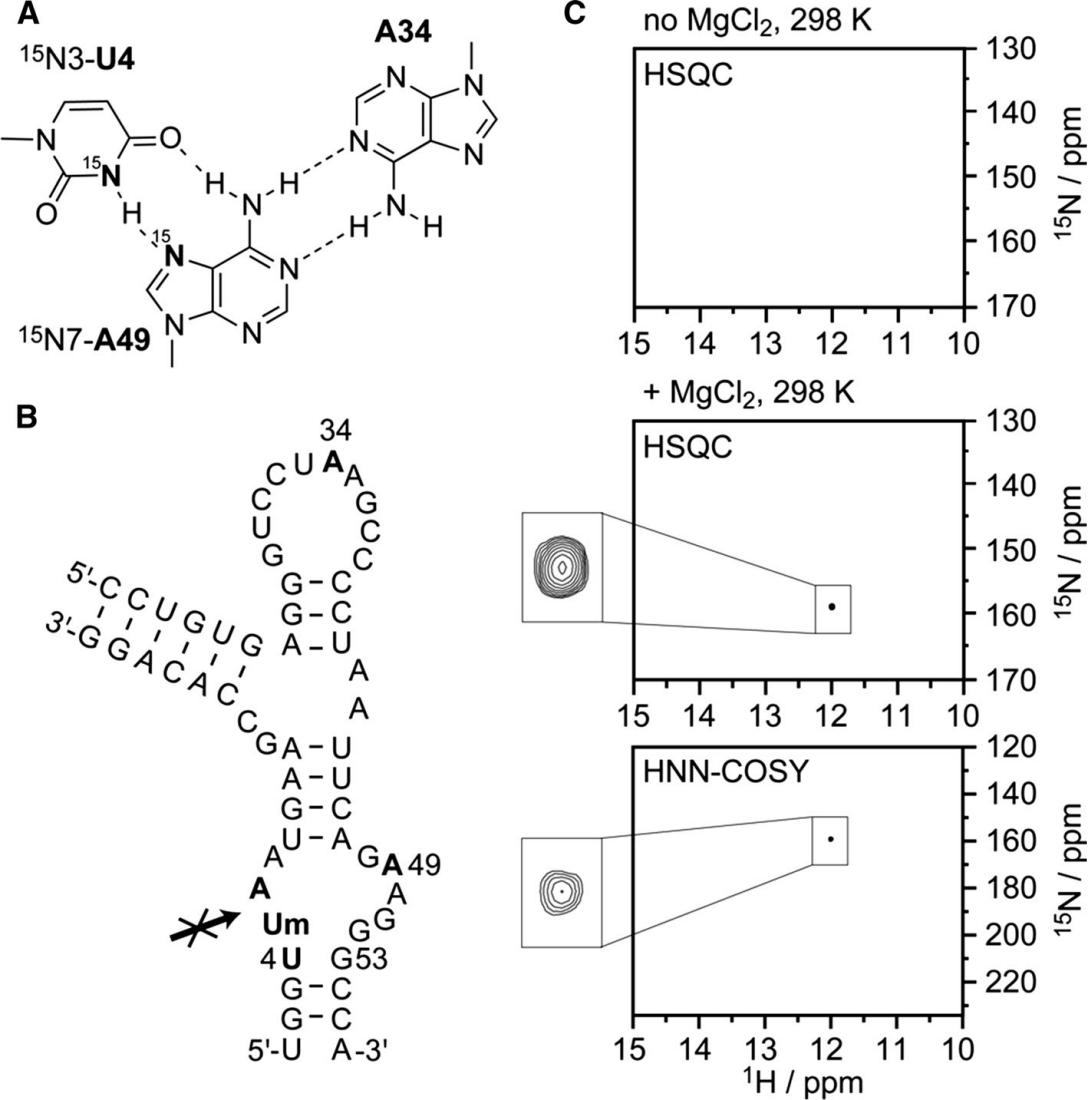
Selective labeling of the U4·A49-A34 base triplet observed in the crystal structure of the *env22* twister ribozyme [8, 19]. **a** Chemical structures of the base triplet with ^15^N-labeled nucleobases to probe the *cis* Watson–Crick/Hoogsteen U4·A49 interaction. **b** Secondary structure of the non-cleavable bimolecular twister ribozyme construct used for the NMR study (Um: 2′-OCH_3_ uridine). **c** HSQC and HNN COSY NMR spectra; conditions: *c*(RNA) = 0.4 mM; 100 mM KCl, 10 mM Na cacodylate, pH 7.0, H_2_O/D_2_O 9/1, 298 K; addition of 2 mM MgCl_2_ as indicated

Our motivation to synthesize ^15^N(7) adenosine phosphoramidite **12** refers to the structural distinctions that have been found recently in the crystal structures of the twister ribozyme [8, 18–20]. Interestingly, while in the *O. sativa* twister ribozyme, the phylogenetically highly conserved four-base pair stem P1 was formed as predicted [18], in the *env22* ribozyme; only two base pairs of stem P1 were observed; two nucleotides (U1 and U4) fold back to the core of the ribozyme and were involved in triplet interactions [8, 19]. One of these triplets (U4·A49-A34) very close to the active site of the ribozyme (Fig. 1) can be considered to affect the active site conformation to support phosphodiester cleavage. It was this base triplet that we intended to verify in solution by direct monitoring of the A49-N(7)·HN(3)-U4 hydrogen bond interaction, applying HNN COSY NMR experiments. We, therefore, resorted to the same bimolecular twister RNA construct that we previously designed for p*K*_a_ determination of the putative general acid A6 at the cleavage site [19]. This time, however, the RNAs were synthesized with ^15^N(3)-labeled U4 and ^15^N(7)-labeled A49.

Indeed, in the HSQC NMR spectra, a resonance appeared at 161.5 ppm in the presence of saturating concentration of Mg^2+^, indicating the reduced exchange of the H-^15^N(3)-U4 with the solvent and thereby supporting a defined base pair interaction. Unfortunately, our attempts to verify this interaction directly by a correlation signal between H-^15^N(3)-U4 and ^15^N(7)-A49 in the HNN COSY experiments failed so far [21, 22]. The ^15^N(3) chemical shift value observed for U4 (158.8 ppm) is in the typical ppm range (between 155 and 165 ppm) of Watson–Crick UA, *trans* Watson–Crick/Hoogsteen UA, and also wobble UG pairs that were observed also in other RNAs [21, 23, 24]. The H-^15^N(3)-U4 chemical shift value observed for U4 (11.92 ppm) is slightly higher compared to typical H-^15^N(3)-U shifts in GU wobble base pairs (11.5–11.8 ppm) but also not in the typical range of reversed Hoogsteen AU pairs (12.5–13.0 ppm) [21, 23, 24]. At this time, we, therefore, cannot completely exclude alternative U4·G53 wobble base pair formation within stem P1 in solution although we favor the view of a preference for triplet formation. Further experiments and experimental designs are needed to answer this particular question in the context of twister ribozyme folding.

## Conclusion

We developed a robust synthesis of a ^15^N(7)-adenosine building block for automated RNA solid-phase synthesis. The labeled nucleoside was generated via a silyl-Hilbert-Johnson reaction of ^15^N(7)-hypoxanthine and protected ribofuranose. To transform the resulting inosine to adenosine, the reaction sequence included a very mild azido-to-acetamido transformation using thioacetic acid. To demonstrate a potential application, we incorporated ^15^N(7)-adenosine into RNA to probe a specific base triplet interaction that was observed in the crystal structures of the twister ribozyme by NMR spectroscopy in solution.

## Experimental

Reagents were purchased in the highest available quality from commercial suppliers (Sigma Aldrich, Acros) and used without further purification. Sodium ^15^N-nitrite (98 atom % ^15^N) was obtained from Sigma Aldrich. Moisture sensitive reactions were carried out under argon atmosphere. ^1^H and ^13^C spectra were recorded on a Bruker DRX 300 MHz spectrometer. Chemical shifts (*δ*) are reported relative to tetramethylsilane (TMS) referenced to the residual proton signal of the deuterated solvent (DMSO-*d*_*6*_: 2.50 ppm for ^1^H spectra and 39.52 ppm for ^13^C spectra; CDCl_3_: 7.26 ppm for ^1^H spectra and 77.16 ppm for ^13^C spectra). The following abbreviations were used to denote multiplicities: *s* singlet, *d* doublet, *t* triplet, *m* multiplet, *b* broad. Signal assignments are based on ^1^H-^1^H-COSY and ^1^H-^13^C-HSQC experiments. MS experiments were performed on a Finnigan LCQ Advantage MAX ion trap instrumentation (Thermo Fisher Scientific) with an electrospray ion source. Samples were analyzed in the positive- or negative-ion mode. Reaction control was performed via analytical thin-layer chromatography (TLC, Macherey–Nagel) with fluorescent indicator. Spots were further visualized using cerium molybdate or anisaldehyde staining reagents. Column chromatography was carried out on silica gel 60 (70–230 mesh).

### [^15^N(7)]-2′,3′,5′-Tri-*O*-benzoylinosine (**6**, C_31_H_24_N_3_^15^NO_8_)

^15^N(7)-Hypoxanthine **5** (2.00 g, 14.6 mmol) and 7.36 g 1-*O*-acetyl-2,3,5-*O*-tribenzoylribofuranose (14.6 mmol) were suspended in 100 cm^3^ of dry toluene. *N*,*O*-Bis(trimethylsilyl)acetamide (10.7 cm^3^, 43.8 mmol) was added and the suspension refluxed for 30 min upon which a clear yellow solution formed. At this point 7.9 cm^3^ trimethylsilyl triflate (43.8 mmol) was added and refluxing was continued for 45 min. All volatiles were evaporated and the residue was dissolved in 350 cm^3^ of dichloromethane and 150 cm^3^ of saturated sodium bicarbonate solution. The organic layer was washed with 150 cm^3^ of brine, dried over Na_2_SO_4_, filtered, and evaporated. The crude product was purified by silica gel column chromatography, eluting with 0–5% methanol in dichloromethane. Compound **6** was obtained as an off-white foam. Yield: 4.72 g (56%); *R*_*f*_ = 0.20 (MeOH/CH_2_Cl_2_ 5:95); ^1^H NMR (300 MHz, DMSO-*d*_*6*_): *δ* = 4.64–4.88 (m, 3H, H-C(4′), 2 × H-C(5′)), 6.19 (t, *J* = 5.9 Hz, 1H, H-C(3′)), 6.40 (t, *J* = 5.3 Hz, 1H, H-C(2′)), 6.57 (d, *J* = 4.6 Hz, 1H, H-C(1′)), 7.41–7.53 (m, 6H, H-C(ar, Bz)), 7.61–7.69 (m, 3H, H-C(ar, Bz)), 7.88–8.00 (m, 7H, H-C(2), H-C(ar, Bz)), 8.38 (d, *J* ^1^_H_^15^_N_ = 12.0 Hz, 1H, H-C(8)), 12.49 (br s, 1H, H-N(1)) ppm; ^13^C NMR (75 MHz, DMSO-*d*_*6*_): *δ* = 63.25 (C(5′)), 70.69 (C(3′)), 73.31 (C(2′)), 79.31 (C(4′)), 86.51 (C(1′)), 125.04 (C(ar)), 128.30 (C(ar, Bz)), 128.75 (C(ar, Bz)), 129.29 (C(ar, Bz)), 129.37 (C(ar, Bz)), 133.52 (C(ar, Bz)), 133.89 (C(ar, Bz)), 134.00 (C(ar, Bz)), 139.78 (C(8)), 146.15 (C(2)), 147.89 (C(ar)), 156.39 (C(ar)), 164.50 (CO(Bz)), 164.68 (CO(Bz)), 165.42 (CO(Bz)) ppm; ESI–MS: *m/z* = 581.88 ([M + H]^+^).

### [^15^N(*7*)]-6-Azido-9-(2′,3′,5′-tri-O-benzoylribofuranosyl)purine (**7**, C_31_H_23_N_6_^15^NO_7_)

Compound **6** (4.67 g, 8.03 mmol) was dissolved in 64 cm^3^ of dry DMF and 5.51 g cesium carbonate (16.9 mmol) and 7.47 g (benzotriazol-1-yloxy)tris(dimethylamino)phosphonium hexafluorophosphate (16.9 mmol) were subsequently added. The yellowish suspension was stirred for 30 min at room temperature, at which point 1.10 g sodium azide (16.9 mmol) was added. The reaction mixture was stirred for another 2 h and then concentrated in vacuo. The resulting slurry was taken up in 200 cm^3^ of water and extracted with four 100 cm^3^ portions of ethyl acetate. The combined organic phases were washed twice with half saturated sodium chloride solution and finally with brine. The ethyl acetate phase was dried over sodium sulfate and evaporated. The crude product was purified by silica gel column chromatography, eluting with 20–60% ethyl acetate in *n*-hexane. Yield: 3.39 g white foam (70%); *R*_*f*_ = 0.50 (ethyl acetate/*n*-hexane 1:1); ^1^H NMR (300 MHz, DMSO-*d*_*6*_): *δ* = 4.70–4.90 (m, 2H, 2 × H-C(5′)), 4.97–4.99 (m, 1H, H-C(4′)), 6.27 (t, *J* = 5.9 Hz, 1H, H-C(3′)), 6.47 (t, *J* = 10.5 Hz, 1H, H-C(2′)), 6.86 (d, *J* = 4.4 Hz, 1H, H-C(1′)), 7.43–7.50 (m, 6H, H-C(ar, Bz)), 7.59–7.69 (m, 3H, H-C(ar, Bz)), 7.90–7.97 (m, 6H, H-C(ar, Bz)), 8.96 (d, *J* ^1^_H_^15^_N_ = 12.2 Hz, H-C(8)), 10.03 (s, 1H, H-C(2)) ppm; ^13^C NMR (75 MHz, DMSO-*d*_*6*_): *δ* = 63.34 (C(5′)), 70.74 (C(3′)), 73.70 (C(2′)), 79.72 (C(4′)), 87.12 (C1′)), 121.02 (C(ar)), 128.26 (C(ar, Bz)), 128.53 (C(ar, Bz)), 128.68 (C(ar, Bz)), 128.77 (C(ar, Bz)), 128.92 (C(ar, Bz)), 129.21 (C(ar, Bz)), 129.41 (C(ar, Bz)), 133.49 (C(ar, Bz)), 133.94 (C(ar, Bz)), 134.05 (C(ar, Bz)), 136.25 (C(2)), 141.40 (C(ar)), 143.76 (C(8)), 164.50 (CO(Bz)), 164.67 (CO(Bz)), 165.39 (CO(Bz)) ppm.

### [^15^N(7)]-N^6^-Acetyl-2′,3′,5′-tri-*O*-benzoyladenosine (**8**, C_33_H_27_N_4_^15^NO_8_)

Compound **7** (3.22 g, 5.31 mmol) was dissolved in 50 cm^3^ of dry dichloromethane and 3.15 cm^3^ 2,6-lutidine (27 mmol) and 2.45 cm^3^ thioacetic acid (32 mmol) were added. The solution was stirred at room temperature for 21 h. All volatiles were evaporated and the residue dissolved in dichloromethane. The organic layer was washed with saturated sodium bicarbonate solution, 5% citric acid, and brine. After drying over Na_2_SO_4_ and evaporation, a dark red oil was obtained. The product was isolated by silica gel column chromatography, eluting from 0 to 3% methanol in dichloromethane. Yield: 2.77 g (84%) of compound **8** as a white foam. *R*_*f*_ = 0.35 (MeOH/CH_2_Cl_2_ 5:95); ^1^H NMR (300 MHz, DMSO-*d*_*6*_): *δ* = 2.27 (s, 3H, C(6)-NHCOC*H*_3_), 4.65–4.92 (m, 3H, H-C(4′), 2 × H-C(5′)), 6.30 (t, *J* = 5.9 Hz, 1H, H-C(3′)), 6.54 (t, *J* = 5.3 Hz, 1H, H-C(2′)), 6.68 (d, *J* = 4. 6 Hz, 1H, H-C(1′)), 7.41–7.52 (m, 6H, H-C(ar, Bz)), 7.61–7.68 (m, 3H, H-C(ar, Bz)), 7.88–7.90 (m, 2H, H-C(ar, Bz)), 7.94–8.00 (m, 4H, H-C(ar, Bz)), 8.53 (s, 1H, H-C(2)), 8.72 (d, *J* ^1^_H_^15^_N_ = 12.1 Hz, 1H, H-C(8)), 10.74 (s, 1H, C(6)-N*H*COCH_3_) ppm; ^13^C NMR (75 MHz, DMSO-*d*_*6*_): *δ* = 24.94 (C(6)-NHCO*C*H_3_), 63.75 (C(5′)), 71.27 (C(3′)), 73.61 (C(2′)), 79.89 (C(4′)), 87.18 (C(1′)), 124.43 (C(ar, Bz)), 128.92 (C(ar, Bz)), 129.33 (C(ar, Bz)), 129.88 (C(ar, Bz)), 129.97 (C(ar, Bz)), 134.08 (C(ar, Bz)), 134.48 (C(ar, Bz)), 144.20 (C(8)), 150.43 (C(ar)), 151.94 (C(ar)), 152.43 (C(2)), 165.11 (CO(Bz)), 165.29 (CO(Bz)), 166.02 (CO(Bz)), 169.46 (C(6)-NH*C*OCH_3_) ppm; ESI–MS: *m/z* = 622.99 ([M + H]^+^).

### [^15^N(7)]-N^6^-Acetyladenosine (**9**, C_12_H_15_N_4_^15^NO_5_)

Compound **8** (2.77 g, 4.45 mmol) was dissolved in 37 cm^3^ of pyridine/ethanol 1:1 and cooled in an icebath. Then 46 cm^3^ of 2 M aqueous sodium hydroxide and ethanol 1:1 was rapidly added and the mixture stirred vigorously for 40 min. The reaction was neutralized with Dowex 50WX-8 (pyridinium form) and filtered. The filtrate was evaporated to yield a slightly red residue which was triturated under ether and then dichloromethane. The product was obtained as an off-white solid. Yield: 1.31 g (95%); *R*_*f*_ = 0.40 (MeOH/CH_2_Cl_2_ 2:8); ^1^H NMR (300 MHz, DMSO-*d*_*6*_): *δ* = 2.26 (s, 3H, C(6)-NHCOC*H*_3_), 3.57–3.71 (m, 2H, 2 × H-C(5′)), 3.98 (s, 1H, H-C(4′)), 4.19 (s, 1H, H-C(3′)), 4.63 (s, 1H, H-C(2′)), 6.02 (d, *J* = 5.1 Hz, 1H, H-C(1′)), 8.66 (s, 1H, H-C(2)), 8.71 (d, *J* ^1^_H_^15^_N_ = 12.1 Hz, 1H, H-C(8)), 10.74 (s, 1H, C(6)-N*H*COCH_3_) ppm; ^13^C NMR (75 MHz, DMSO-*d*_*6*_): *δ* = 24.92 (C(6)-NHCO*C*H_3_), 61.91 (C(5′)), 70.95 (C(3′)), 74.29 (C(2′)), 86.34 (C(4′)), 88.26 (C(1′)), 124.17, 129.85, 143.42 (C(8)), 150.15, 152.19 (C(2)), 169.58 (C(6)-NH*C*OCH_3_) ppm.

### [^15^N(7)]-N^6^-Acetyl-5′-*O*-(4,4′-dimethoxytrityl)adenosine (**10**, C_33_H_33_N_4_^15^NO_7_)

Compound **9** (1.27 g, 4.10 mmol) and 75 mg 4-(dimethylamino)pyridine (0.61 mmol) were dried over P_2_O_5_ under high vacuum for 2 h and suspended in 30 cm^3^ of dry pyridine. DMT-Cl (1.60 g, 4.71 mmol) was added in four portions and the resulting solution stirred at room temperature. After 7.5 h, the reaction was quenched with methanol and all volatiles were evaporated. The residue was dissolved in dichloromethane and 5% citric acid. The organic layer was separated and washed with 5% citric acid, saturated sodium bicarbonate solution, and brine, dried over Na_2_SO_4_, filtered, and evaporated. The product was isolated by silica gel column chromatography, eluting with 0–7% methanol in dichloromethane. Yield: 1.49 g (59%) of compound **10** as a white foam. *R*_*f*_ = 0.50 (MeOH/CH_2_Cl_2_ 1:9); ^1^H NMR (300 MHz, DMSO-*d*_*6*_): *δ* = 2.27 (s, 3H, C(6)-NHCOC*H*_3_), 3.25 (d, *J* = 4.1 Hz, 2H, 2 × H-C(5′)), 3.72 (s, 6H, 2 × CH_3_O(DMT)), 4.09–4.14 (m, 1H, H-C(4′)), 4.33–4.38 (m, 1H, H-C(3′)), 4.75–4.80 (m, 1H, H-C(2′)), 5.27 (d, *J* = 6.0 Hz, 1H, HO-C(3′)), 5.61 (d, *J* = 5.6 Hz, 1H, HO-C(2′)), 6.05 (d, *J* = 4.6 Hz, 1H, H-C(1′)), 6.80–6.85 (m, 4H, H-C(ar, DMT)), 7.19–7.27 (m, 7H, H-C(ar, DMT)), 7.35–7.37 (m, 2H, H-C(ar, DMT)), 8.58 (d, *J* ^1^_H_^15^_N_ = 12.2 Hz, 1H, H-C(8)), 8.59 (s, 1H, H-C(2)), 10.70 (s, 1H, C(6)-N*H*COCH_3_) ppm; ^13^C NMR (75 MHz, DMSO-*d*_*6*_): *δ* = 24.95 (C(6)-NHCO*C*H_3_), 55.50 (2 × CH_3_O(DMT)), 64.29 (C(5′)), 70.94 (C(3′)), 73.57 (C(2′)), 83.88 (C(4′)), 86.10, 88.81 (C1′)), 113.72 (C(ar, DMT)), 124.27 (C(ar, DMT)), 127.24 (C(ar, DMT)), 128.28 (C(ar, DMT)), 128.35 (C(ar, DMT)), 130.28 (C(ar, DMT)), 136.09, 136.17, 143.56 (C(8)), 145.41, 150.25, 152.25 (C(2)), 158.64, 169.45 (C(6)-NH*C*OCH_3_) ppm.

### [^15^N(7)]-*N*^6^-Acetyl-5′-*O*-(4,4′-dimethoxytrityl)-2′-*O*-(tert-butyldimethylsilyl)adenosine (**11**, C_39_H_47_N_4_^15^NO_7_Si)

Compound **10** (990 mg, 1.62 mmol) was dissolved in 12 cm^3^ of dry THF and 0.30 cm^3^ of dry pyridine (3.56 mmol). Silver nitrate (440 mg, 2.59 mmol) was added and the mixture was stirred vigorously for 30 min in the dark at room temperature. Then, 429 mg *t*BDMS-Cl (2.84 mmol) was added and stirring was continued for 4 h at which point the suspension was filtered through a bed of Celite and evaporated. After aqueous workup with saturated sodium bicarbonate and brine an off-white foam was obtained. Silica gel column chromatography, eluting with 30–70% ethyl acetate in hexane, yielded 474 mg of the 2′-*O*-silyl-isomer **11** (40%), 329 mg of the 3′-*O*-silyl-isomer (28%), and 209 mg of the starting material was recovered. *R*_*f*_ = 0.50 (MeOH/CH_2_Cl_2_ 7:93); ^1^H NMR (300 MHz, DMSO-*d*_*6*_): *δ* = −0.13 (s, 3H, Si-CH_3_), −0.03 (s, 3H, Si-CH_3_), 0.75 (s, 9H, Si-C(CH_3_)_3_), 2.26 (s, 3H, C(6)-NHCOC*H*_3_), 3.30 (m, 2H, 2 × H-C(5′)), 3.72 (s, 6H, 2 × CH_3_O(DMT)), 4.12–4.17 (m, 1H, H-C(4′)), 4.27–4.33 (m, 1H, H-C(3′)), 4.89 (t, *J* = 4.8 Hz, 1H, H-C(2′)), 5.20 (d, *J* = 5.9 Hz, 1H, HO-C(3′)), 6.06 (d, *J* = 4.8 Hz, 1H, H-C(1′)), 6.83–6.86 (m, 4H, H-C(ar, DMT)), 7.20–7.28 (m, 7H, H-C(ar, DMT)), 7.39–7.41 (m, 2H, H-C(ar, DMT)), 8.57 (s, 1H, H-C(2)), 8.59 (d, *J* ^1^_H_^15^_N_ = 12.2 Hz, 1H, H-C(8)), 10.71 (s, 1H, C(6)-N*H*COCH_3_) ppm; ^13^C NMR (75 MHz, DMSO-*d*_*6*_): *δ* = −5.30 (Si-CH_3_), −4.83 (Si-CH_3_), 17.80 (Si-*C*(CH_3_)_3_), 24.38 (C(6)-NHCO*C*H_3_), 25.53 (Si-C(*C*H_3_)_3_), 54.99 (2 × CH_3_O(DMT)), 63.37 (C(5′)), 70.16 (C(3′)), 74.86 (C(2′)), 83.53 (C(4′)), 85.55, 88.22 (C(1′)), 113.13 (C(ar, DMT), 123.53 (C(ar, DMT),126.66 (C(ar, DMT), 127.65 (C(ar, DMT),127.76 (C(ar, DMT), 129.70 (C(ar, DMT), 135.39 (C(ar)), 142.75 (C(8)), 144.82 (C(ar)), 149.62 (C(ar)), 151.65 (C(2)), 158.06 (C(ar)), 168.77(C(6)-NH*C*OCH_3_) ppm.

### [^15^N(7)]-*N*^6^-Acetyl-5′-*O*-(4,4′-dimethoxytrityl)-2′-*O*-(tert-butyldimethylsilyl)adenosine 3′-*O*-(2-cyanoethyl) diisopropylphosphoramidite (**12**, C_48_H_64_N_6_^15^NO_8_PSi)

Compound **11** (500 mg, 0.689 mmol) was dried over P_2_O_5_ under high vacuum for 1 h and dissolved in 7 cm^3^ of dry dichloromethane. 1-Methylimidazole (0.03 cm^3^, 0.34 mmol), 0.48 cm^3^ *N*,*N*-diisopropylethylamine (2.75 mmol), and 0.41 cm^3^ 2-cyanoethyl *N*,*N*-diisopropylchloro phosphoramidite (1.72 mmol) were added and the resulting solution was stirred for 2 h at room temperature. The reaction mixture was diluted with dichloromethane and washed with saturated sodium bicarbonate solution and brine. The organic phase was dried over Na_2_SO_4_, filtered, and evaporated. The diastereomeric products were isolated by column chromatography with 30–80% ethyl acetate/hexane as eluent. Yield: 610 mg (90%) of compound **12** as a white foam. *R*_*f*_ = 0.40, 0.35 (ethyl acetate/*n*-hexane 2:1); ^1^H NMR (600 MHz, CDCl_3_): *δ* = −0.21 (s, Si-CH_3_), −0.20 (s, Si-CH_3_), −0.05 (s, Si-CH_3_), −0.02 (s, Si-CH_3_), 0.76 (s, Si-C(CH_3_)_3_), 1.05 (s, N-CH(C*H*_3_)_2_), 1.06 (s, N–CH(C*H*_3_)_2_), 1.16–1.20 (m, 2 × N-CH(C*H*_3_)_2_), 2.27–2.35 (m, 1H, POCH_2_C*H*_2_CN), 2.60 (s, C(6)-NHCOC*H*_3_), 2.62–2.68 (m, 1H, POCH_2_C*H*_2_CN), 3.31–3.36 (m, H(b)-C(5′)), 3.54–3.69 (m, H(a)-C(5′), 2 × N–C*H*(CH_3_)_2_), 1H POC*H*_2_CH_2_CN), 3.78 (s, 2 x CH_3_O(DMT)), 3.84–3.99 (m, 1H, POC*H*_2_CH_2_CN), 4.36–4.44 (m, 2H, H-C(3′), H-C(4′), 5.03–5.06 (m, 1H, H-C(2′)), 6.01–6.08 (2 × d, 1H, H-C(1′)), 6.80–6.82 (m, 4H, H-C(ar, DMT)), 7.20–7.37 (m, 7H, H-C(ar, DMT)), 7.44–7.48 (m, 2H, H-C(ar, DMT), 8.17–8.22 (2 × d, *J* ^1^_H_^15^_N_ = 11.8 Hz, 1H, H-C(8)), 8.58, 8.59 (2 × s, 1H, H-C(2)), 8.69 (bs, 1H, C(6)-N*H*COCH_3_) ppm; ^13^C NMR (151 MHz, CDCl_3_): *δ* = −5.02 (Si-CH_3_), −4.57 (Si-CH_3_), 17.99 (Si-*C*(CH_3_)_3_), 18.04 (Si-*C*(CH_3_)_3_), 20.19, 20.23, 20.55, 20.60 (POCH_2_ *C*H_2_CN), 24.69, 24.76, 24.81, 24.85, 24.90 (2 × N-CH(*C*H_3_)_2_, C(6)-NHCO*C*H_3_), 25.70, 25.73, 25.78 (Si-C(*C*H_3_)_3_), 43.04, 43.12, 43.49, 43.57 (2 × N-*C*H(CH_3_)_2_), 55.35 (2 × CH_3_O-DMT), 57.67, 57.81, 58.82, 58.93 (PO*C*H_2_CH_2_CN), 63.19 (C(5′)), 63.33 (C(5′)), 72.76, 72.85 (C(3′)), 73.34, 73.40 (C(3′)),74.76 (C(2′)), 75.36 (C(2′)), 83.95 (C(4′)), 84.29 (C(4′)), 86.76 (C(DMT)), 86.90 (C(DMT)), 88.42 (C(1′)), 88.66 (C(1′)), 113.33 (C(ar, DMT)), 117.42, 117.71 (POCH_2_CH_2_ *C*N), 122.16, 122.21, 127.11, 128.02, 128.04, 128.26, 128.37, 130.22, 130.27, 130.30 (C(ar, DMT)), 135.61, 135.65, 135.79, 135.84 (C(ar)), 141.99 (C(8)), 142.01 (C(8)), 144.56, 144.68, 149.25, 151.35 (C(ar)), 152.51 (C(2)), 158.70 (C(6)-NHCO*C*H_3_) ppm; ^31^P NMR (121.5 MHz, CDCl_3_): *δ* = 151.5, 149.8 ppm; HRMS: *m/z* calculated 927.4366 ([M + H]^+^), found 927.4368.

## Electronic supplementary material

Below is the link to the electronic supplementary material.

Supplementary material 1 (PDF 664 kb)
