# Supplementary material for: The synthesis of 15N(7)-Hoogsteen face-labeled adenosine phosphoramidite for solid-phase RNA synthesis
Source: Monatsh Chem. 2016 Dec 8;148(1):149–55. doi: 10.1007/s00706-016-1882-8 (PMC5225212; doi:10.1007/s00706-016-1882-8)
Supplement: Supplementary file 1 — Supplementary material 1 (PDF 664 kb) [file 706_2016_1882_MOESM1_ESM.pdf]

Supporting Information

to

The synthesis of ^{15}N(7)-Hoogsteen face labeled adenosine phosphoramidite for solid-phase RNA synthesis

Sandro Neuner[1] ● Christoph Kreutz[1] ● Ronald Micura[1]

[1]Institute of Organic Chemistry and Center for Molecular Biosciences, University of Innsbruck, Austria

Contents

1. NMR spectra of compound **12** 2

2. MS spectra of compound **12** 3

3. ESI-TOF MS spectra of compound **12** 4

4. Qualitative analysis of regioisomers after nucleosidation of ^{15}N(7)-hypoxanthine and 1-*O*-acetyl-2,3,5-*O*-tribenzoylribofuranose 5

1. ^{1}H-NMR (600 MHz, $CDCl_3$) spectrum of compound **12**:

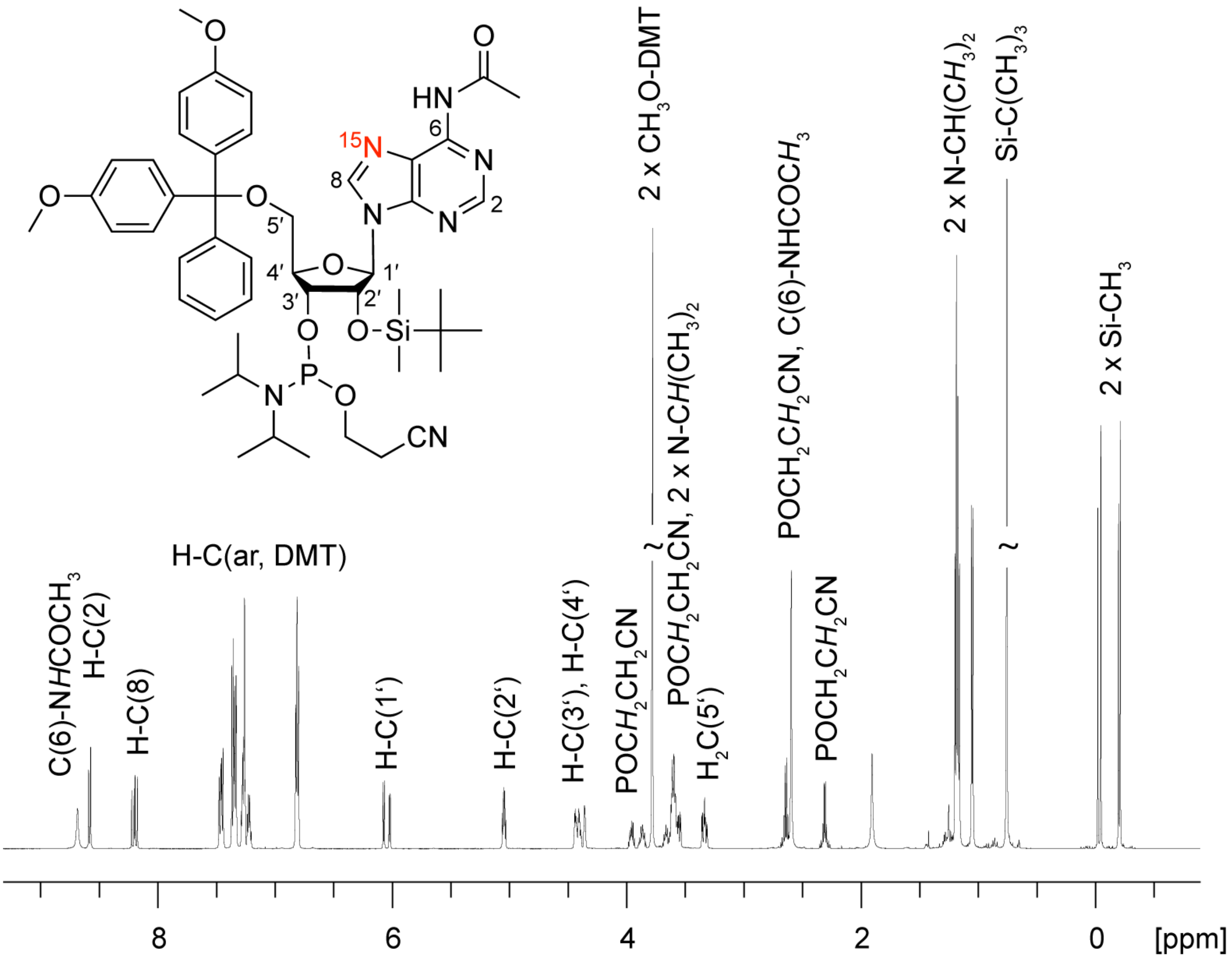

2. ^{13}C-NMR (150 MHz, $CDCl_3$) spectrum of compound **12**:

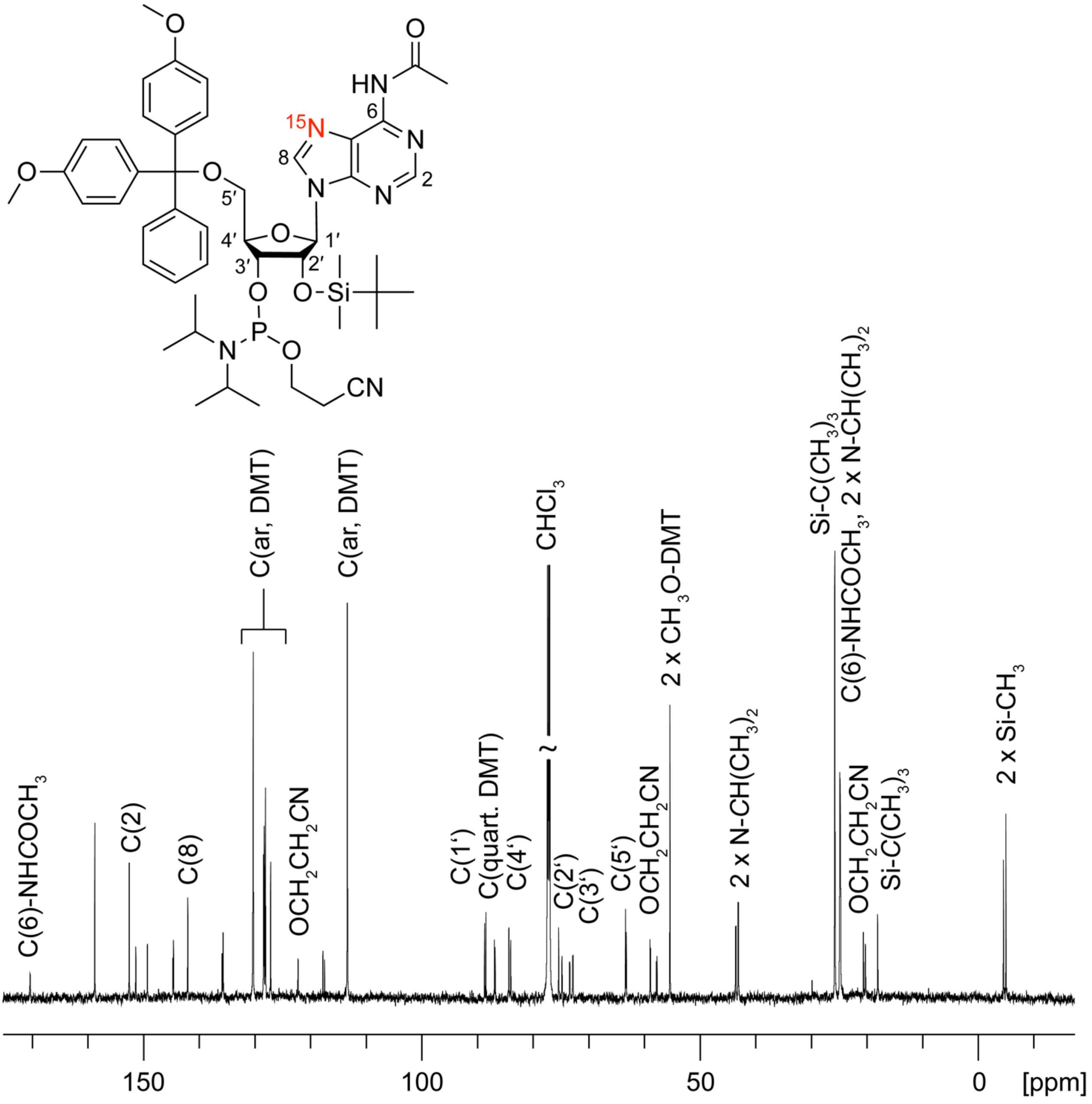

3. ESI-TOF MS spectra of compound 12

Experimental high resolution mass spectrum

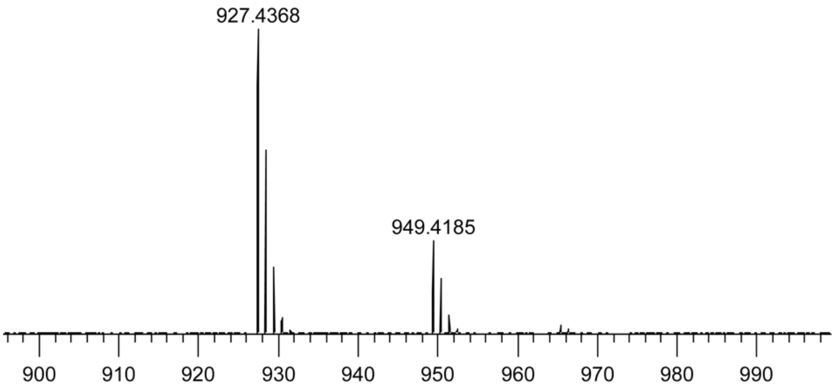

Simulated mass spectra of $M+H^+$ and $M+Na^+$

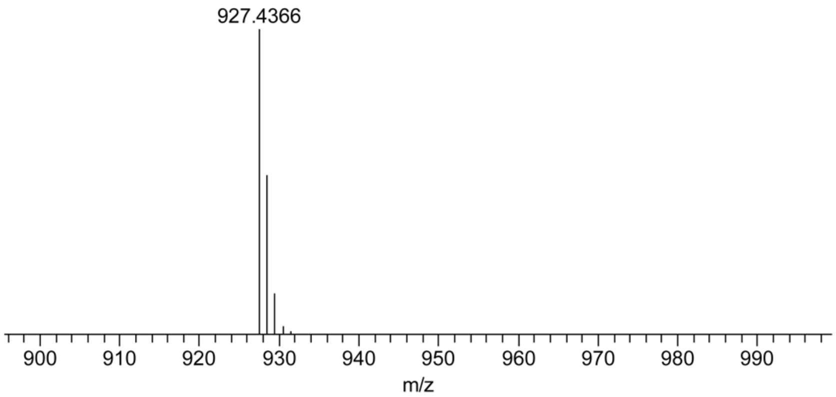

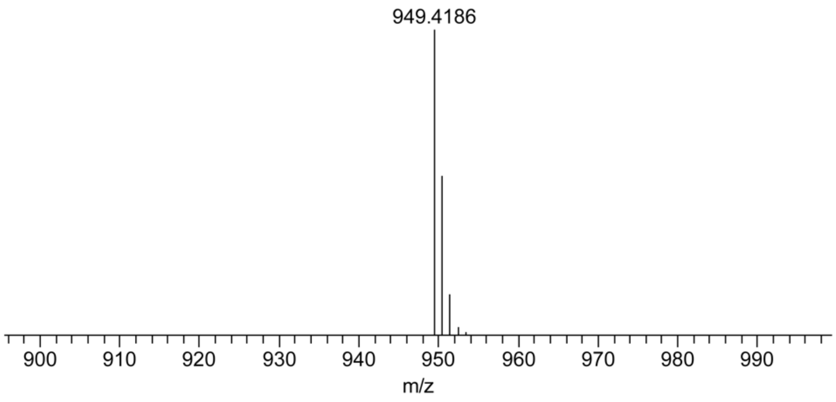

4. Qualitative comparative analysis of regioisomers after nucleosidation of ^{15}N(7)-hypoxanthine (5) and 1-*O*-acetyl-2,3,5-*O*-tribenzoylribofuranose

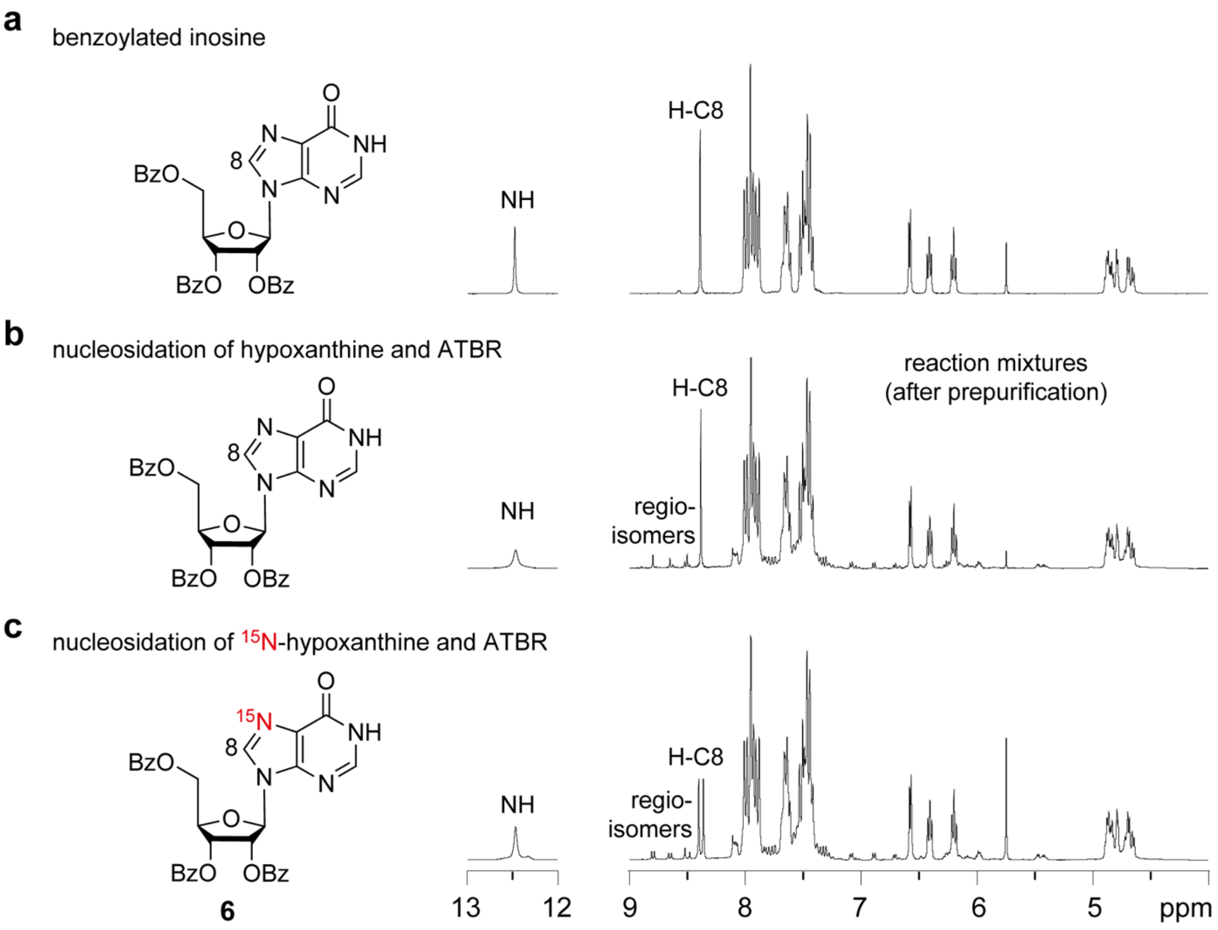

Comparison of ^{1}H-NMR (300 MHz, $CDCl_3$) spectra of inosine derivatives prepared by different routes: **a**) ^{1}H-NMR spectrum of benzoylated inosine obtained from commercial inosine after benzoylation; **b**) ^{1}H-NMR spectrum of benzoylated inosine obtained by *silyl*-Hilbert-Johnson nucleosidation of commercial 1-*O*-acetyl-2,3,5-*O*-tribenzoylribofuranose and hypoxanthine (according to the pathway given in this paper); **c**) ^{1}H-NMR spectrum of benzoylated ^{15}N(7)-inosine **6** obtained by *silyl*-Hilbert-Johnson nucleosidation of commercial 1-*O*-acetyl-2,3,5-*O*-tribenzoylribofuranose and ^{15}N(7)-hypoxanthine (for details see Experimental Part).
